# Supplementary material for: Invariant γδTCR natural killer-like effector T cells in the naked mole-rat
Source: Nat Commun. 2024 May 18;15:4248. doi: 10.1038/s41467-024-48652-z (PMC11102460; doi:10.1038/s41467-024-48652-z)
Supplement: Supplementary file 1 — Supplementary Information [file 41467_2024_48652_MOESM1_ESM.pdf]

Invariant γδTCR natural killer-like effector T cells in the naked mole-rat

Guillem Sanchez Sanchez[1,2,3,4], Stephan Emmrich[5], Maria Georga[7], Ariadni Papadaki[7], Sofia Kossida[7], Andrei Seluanov[5,6], Vera Gorbunova[5,6] and David Vermijlen[1,2,3,4,#]

[1]Department of Pharmacotherapy and Pharmaceutics, Université Libre de Bruxelles (ULB), Brussels, Belgium. [2]Institute for Medical Immunology, Université Libre de Bruxelles (ULB), Gosselies, Belgium. [3]ULB Center for Research in Immunology (U-CRI), Université Libre de Bruxelles (ULB), Belgium. [4]WELBIO Department, WEL Research Institute, Wavre, Belgium. [5]Department of Biology, University of Rochester, Rochester, NY, USA. [6]Department of Medicine, University of Rochester Medical Center and Medicine, University of Rochester, Rochester, NY, USA. [7]IMGT®, the international ImMunoGenetics information system®, Institut de Génétique Humaine (IGH), Centre National de la Recherche Scientifique (CNRS), Université de Montpellier (UM), Montpellier, France.

A

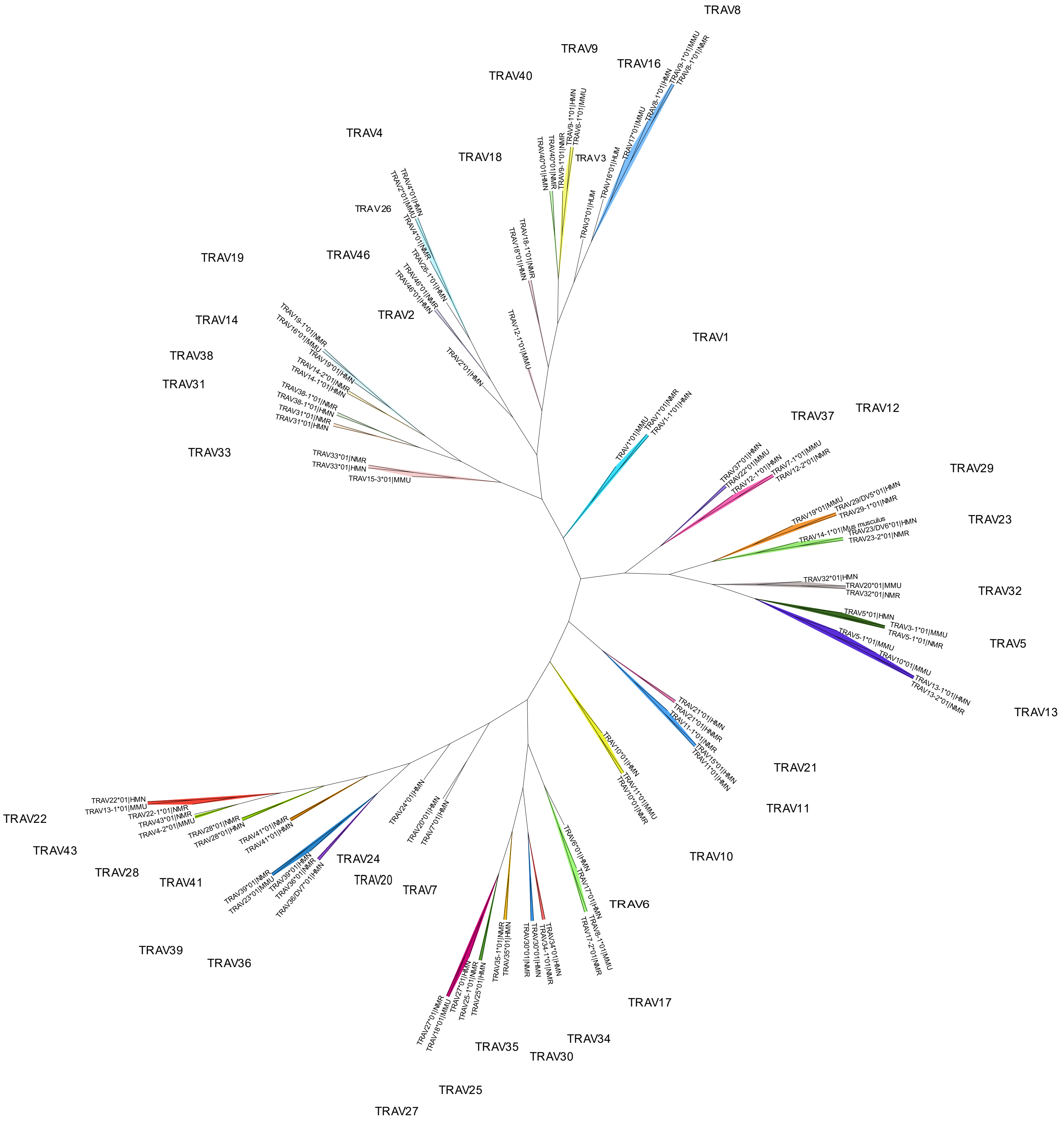

Supplementary Figure 1. (legend: see next page).

B

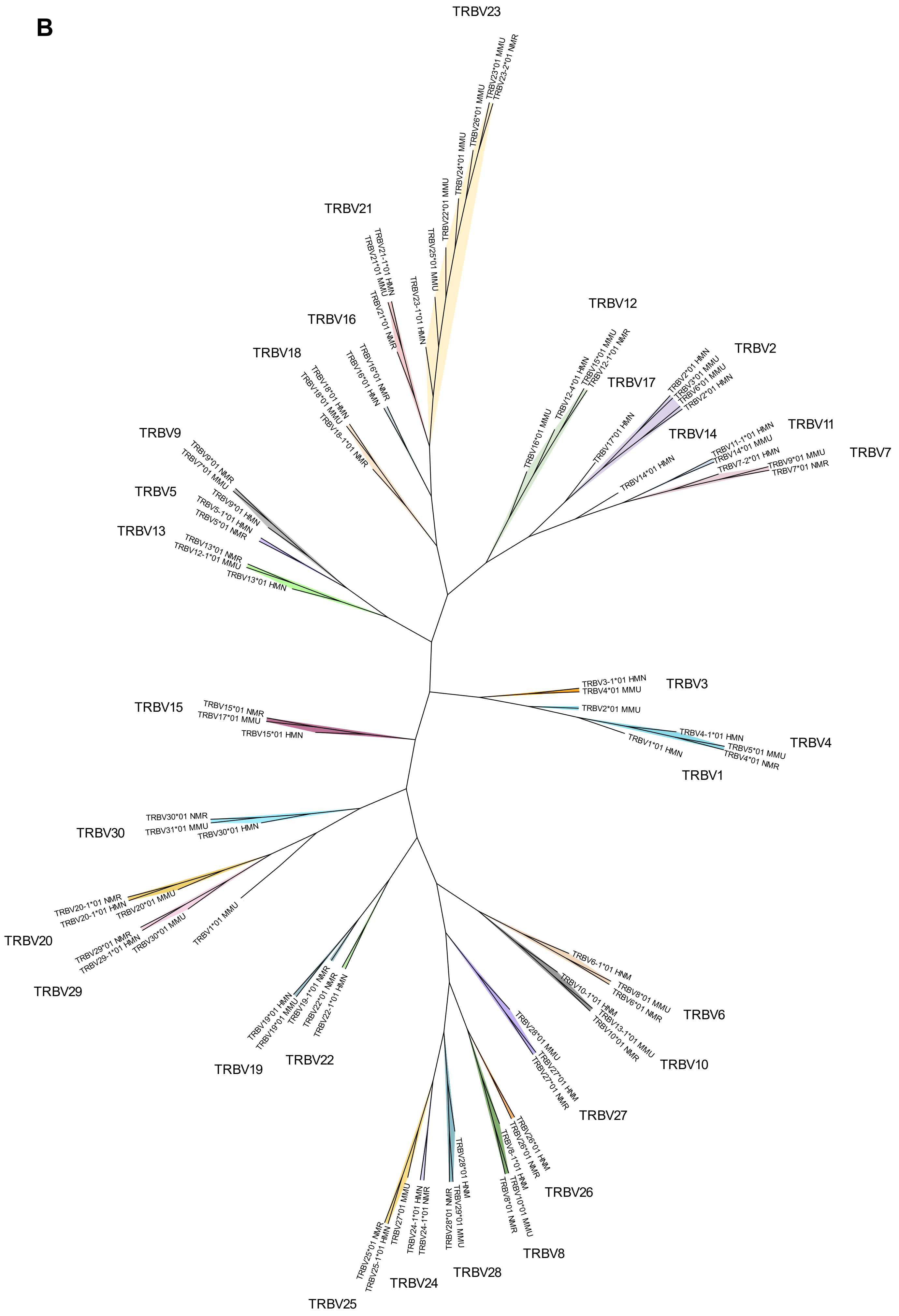

Supplementary Figure 1. (legend: see next page).

Supplementary Figure 1. Unrooted phylogenetic trees of TRAV (A) and TRBV (B) genes. The trees are based on nucleotide sequence alignments, generated with representatives of V-REGION sequences from each subgroup using MAFFT[1]. Visualization of the trees was carried out using iTOL v6[2], with distinct colors representing the different human subgroups and their orthologs. Species abbreviations are as follows: HMN, *Homo sapiens*; MMU, *Mus musculus*; NMR, *Heterocephalus glaber.* Source data are provided as a Source Data file.

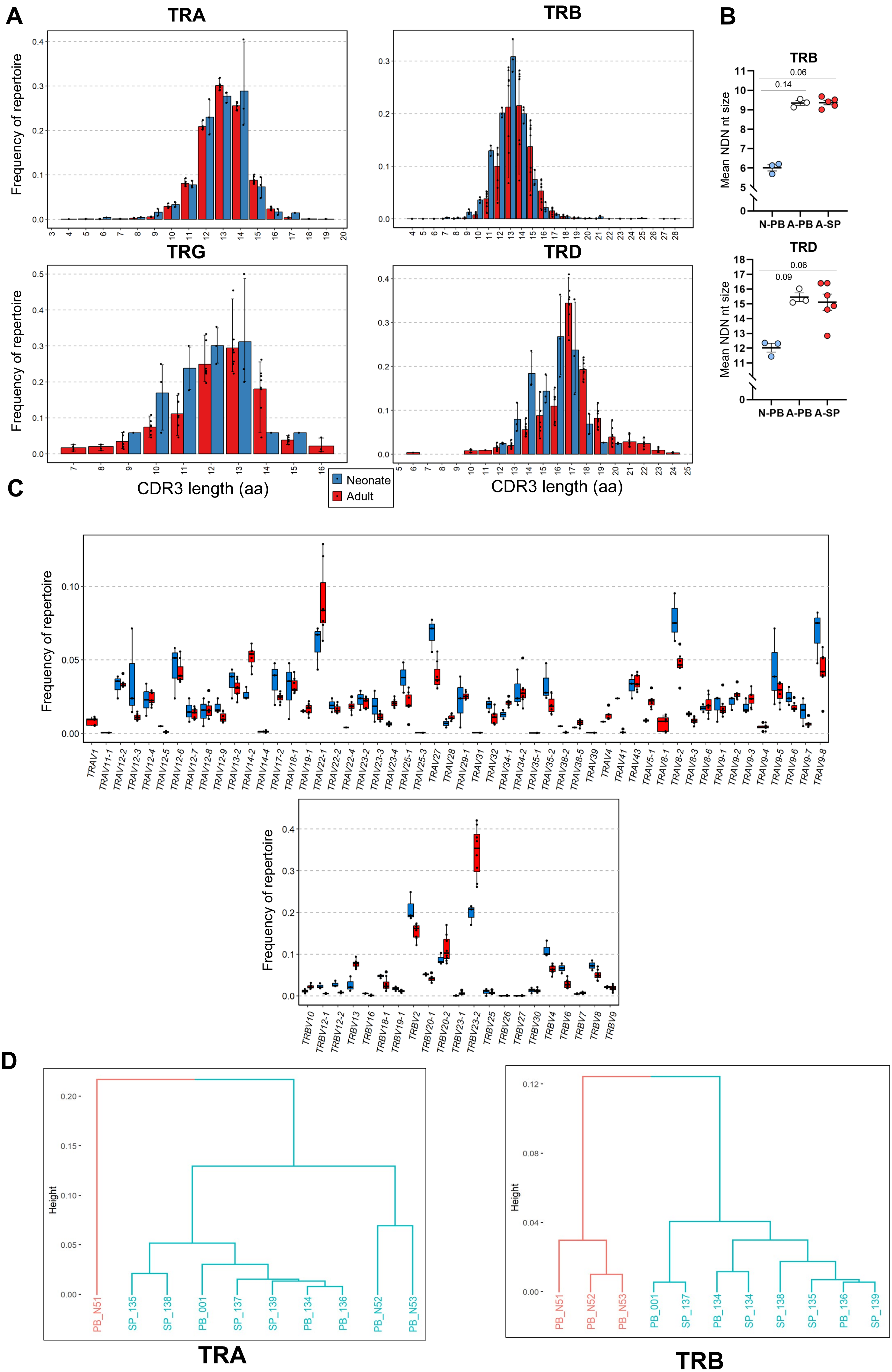

Supplementary Figure 2. (legend: see next page).

Supplementary Figure 2. Analysis of general properties from naked mole-rat TCR repertoire. (A) CDR3 length distribution from the distinct repertoires of neonate blood samples (n=3) and adult blood and spleen samples (n=7-8). (B) Mean NDN size of TRA and TRD repertoire. Blue dots: neonate blood (N-PB, n=3), white dots: adult blood (A-PB, n=3), red dots: adult spleen (A-SP, n=5-6). Each dot represents a naked mole-rat. (C) Boxplots of TRAV (left) and TRBV (right) usage from the distinct repertoires of neonate blood samples (n=3) and adult blood and spleen samples (n=7). (D) Hierarchical clustering of TRAV and TRBV gene usage with Jensen–Shannon divergence used as metric. See **Supplementary Table 1** for more information of each sample ID. (A) The error bars represent the 95% confidence interval around the mean value (height of the bar). (C) Boxplots display the interquartile range (25th to 75th percentile) with the median line. Whiskers extend to the minimum and maximum values within a certain distance from the quartiles, with outliers shown as larger dots. (B) Data analyzed using Kruskal-Wallis with Dunn's multiple tests for pairwise comparisons. (C) Data analyzed using Kruskal-Wallis with Holm correction for pairwise comparisons. Source data are provided as a Source Data file.

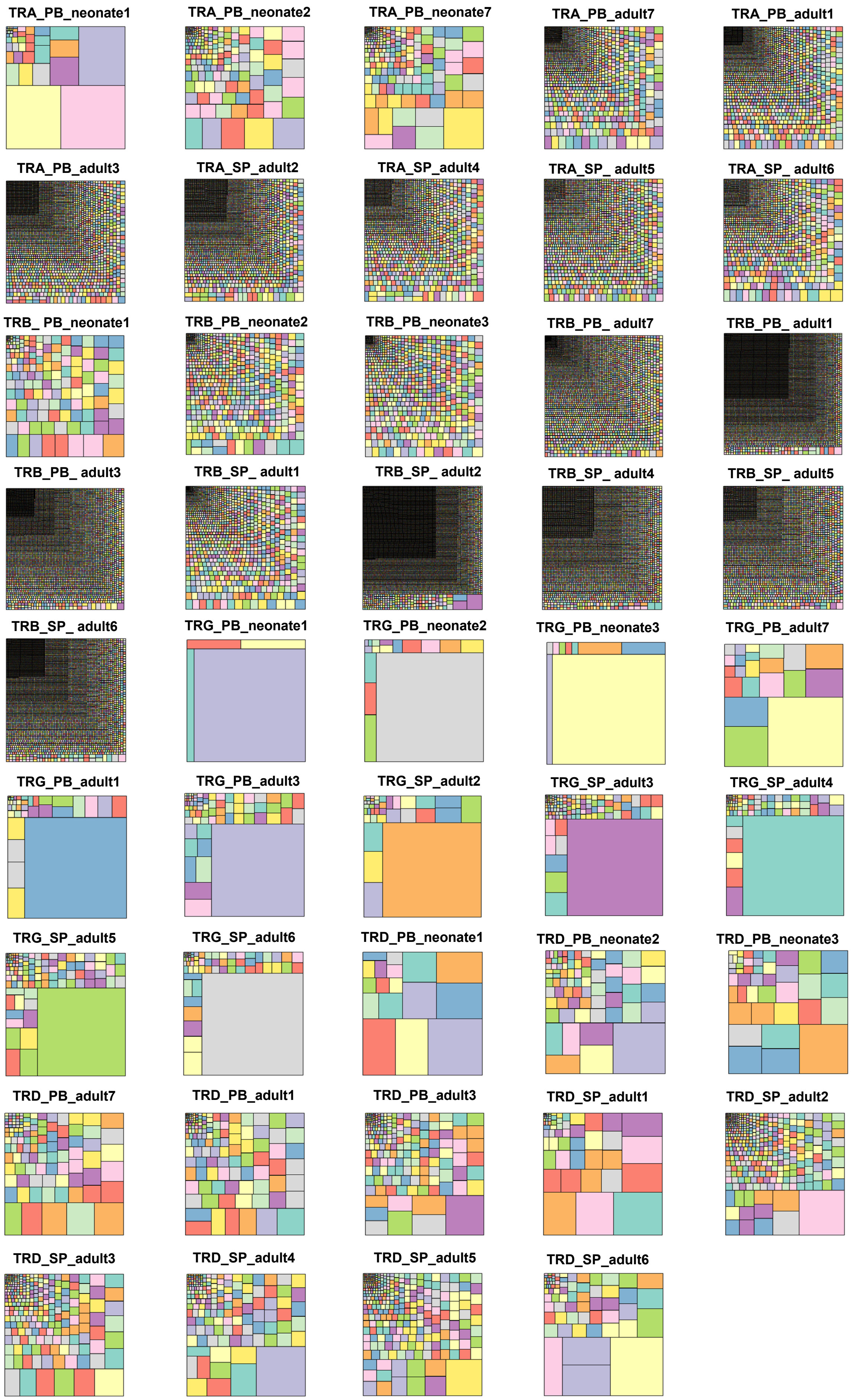

Supplementary Figure 3. Tree map overview of the TRA, TRB, TRG and TRA repertoires of the naked mole-rats used in this study. See **Supplementary Table 1** for sample IDs.

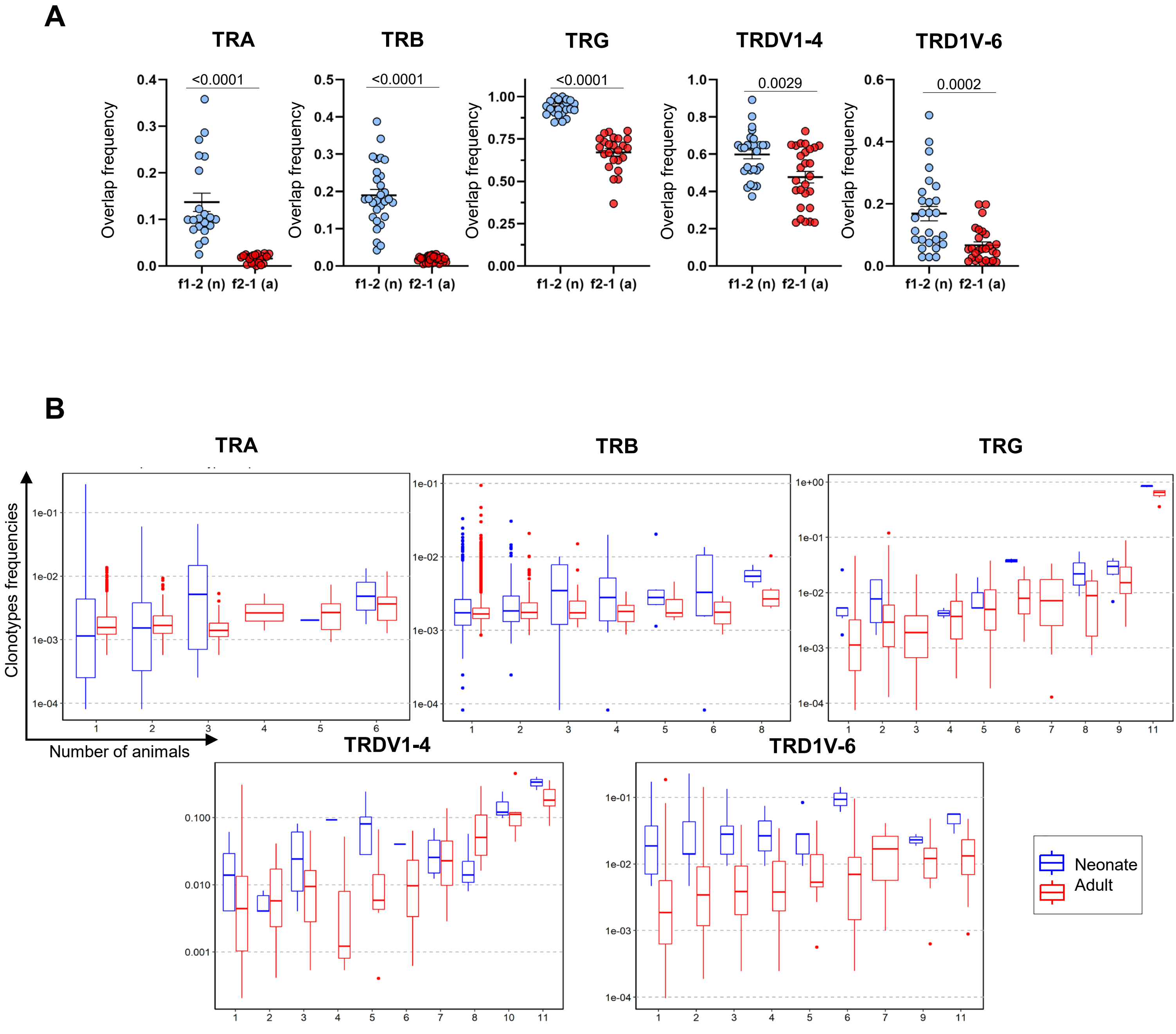

Supplementary Figure 4. TCR sharing and publicity values. (A) Plots displaying the repertoire space occupied by shared CDR3 sequences between neonate (f1-2) (n=3) and adult (f2-1) blood (n=3) and spleen (n=5-6) samples combined in a single group. (B) Boxplots showing the frequencies of all the clonotypes of the distinct repertoires. Clonotypes are grouped depending on their number of occurrences in the different animals. Source data are provided as a Source Data file.

A

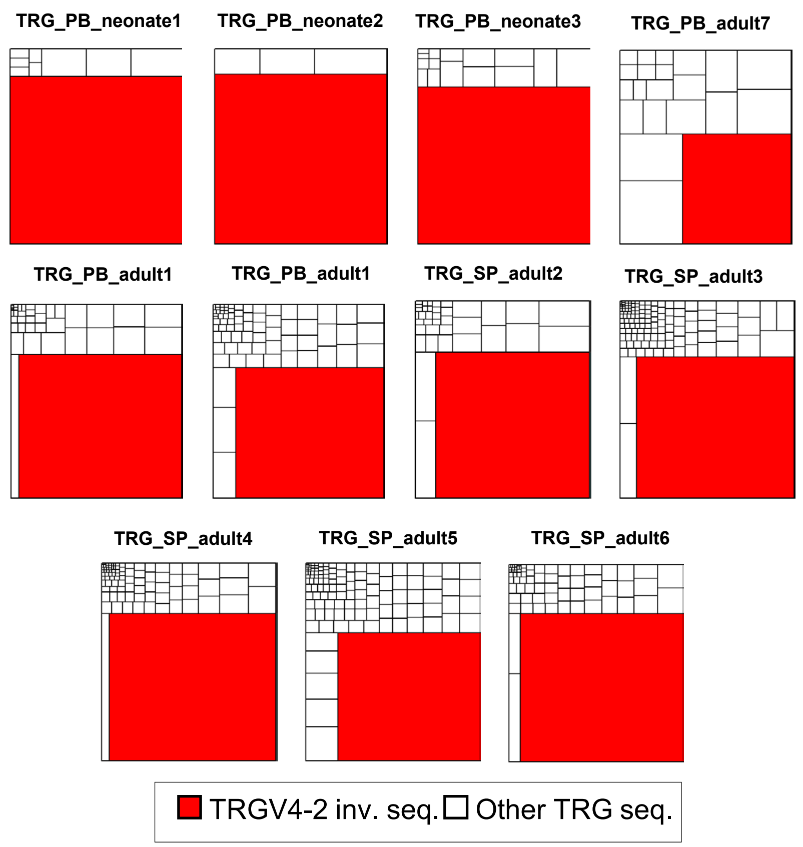

B

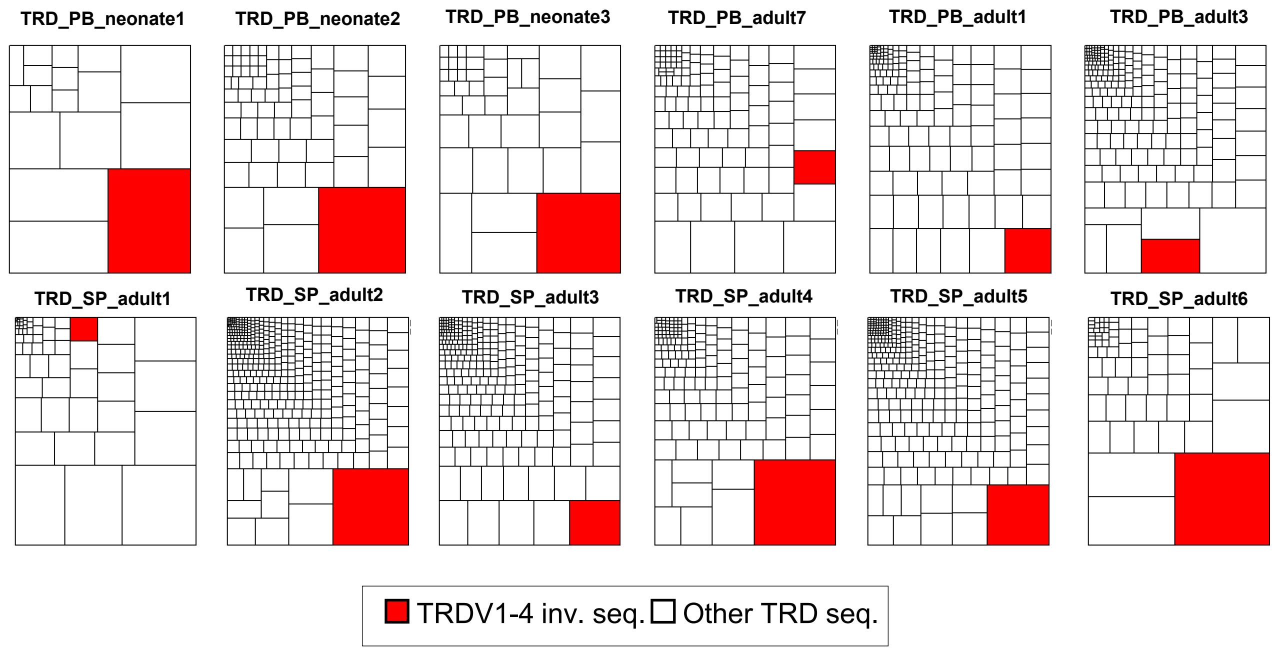

Supplementary Figure 5. Tree maps Illustrating Abundance of invariant γδ TCR sequences in neonate and adult animals (A) Tree maps representing the TRG repertoire in neonate and adult animals, highlighting the prevalence of the TRGV4-2-containing invariant CDR3 sequence (see also **Fig. 4A (left panel) and Fig. 4B**) shown in red. (B) Tree maps illustrating the TRD repertoire in neonate and adult animals, indicating in red the abundance of the TRDV1-4-containing invariant CDR3 sequence (see also **Fig. 4A (right panel) and Fig. 4C)**. (A, B) Tree maps are the same as the ones depicted in **Supplementary Fig. 3.**

See **Supplementary Table 1** for more information regarding each sample ID.

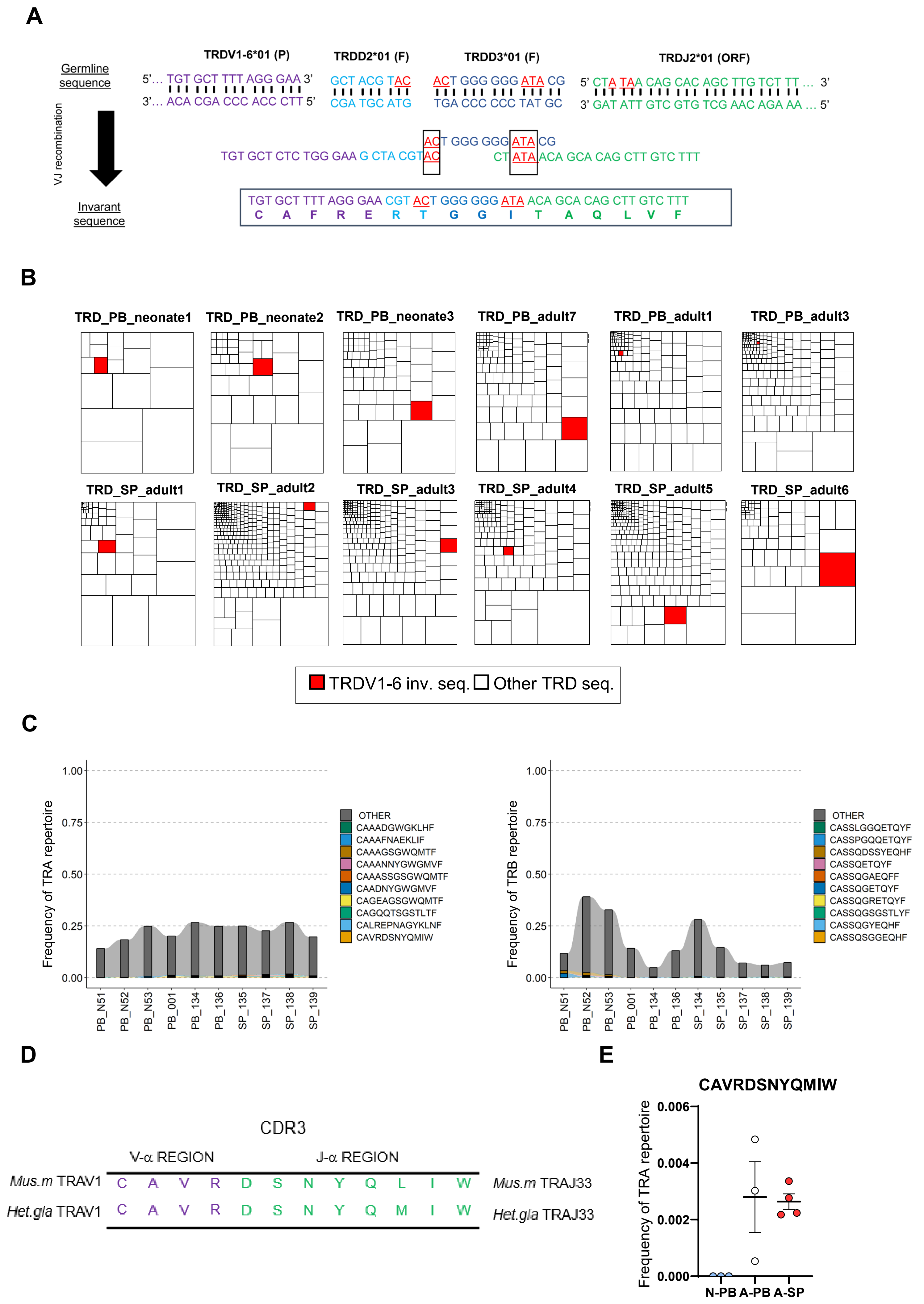

Supplementary Figure 6. (legend: see next page).

Supplementary Figure 6. The naked mole-rat possesses other invariant TCR sequences. (A) Recombination using short homology repeats between *TRDV1-6, TRDD2*, *TRDD3* and *TRDJ2*. (B) Tree maps illustrating the TRD repertoire in neonate and adult animals, indicating in red the abundance of the TRDV1-6-containing invariant CDR3 sequence. (C) Alluvial plots displaying the frequency of top 10 shared TRA (left) and TRB (right) sequences in naked mole-rats. (B, C) See **Supplementary Table 1** for more information regarding each sample ID. (D) Alignment of invariant mouse *TRAV1-TRAJ33* amino acid MAIT sequences from mouse (top) and naked mole-rat (bottom). (E) Frequency of MAIT-like CAVRDSNYQMIW sequence of total repertoire of naked mole-rat animals. N-PB: neonate blood, n=3; A-PB: adult blood, n=3; A-SP: adult spleen, n=4. Source data are provided as a Source Data file.

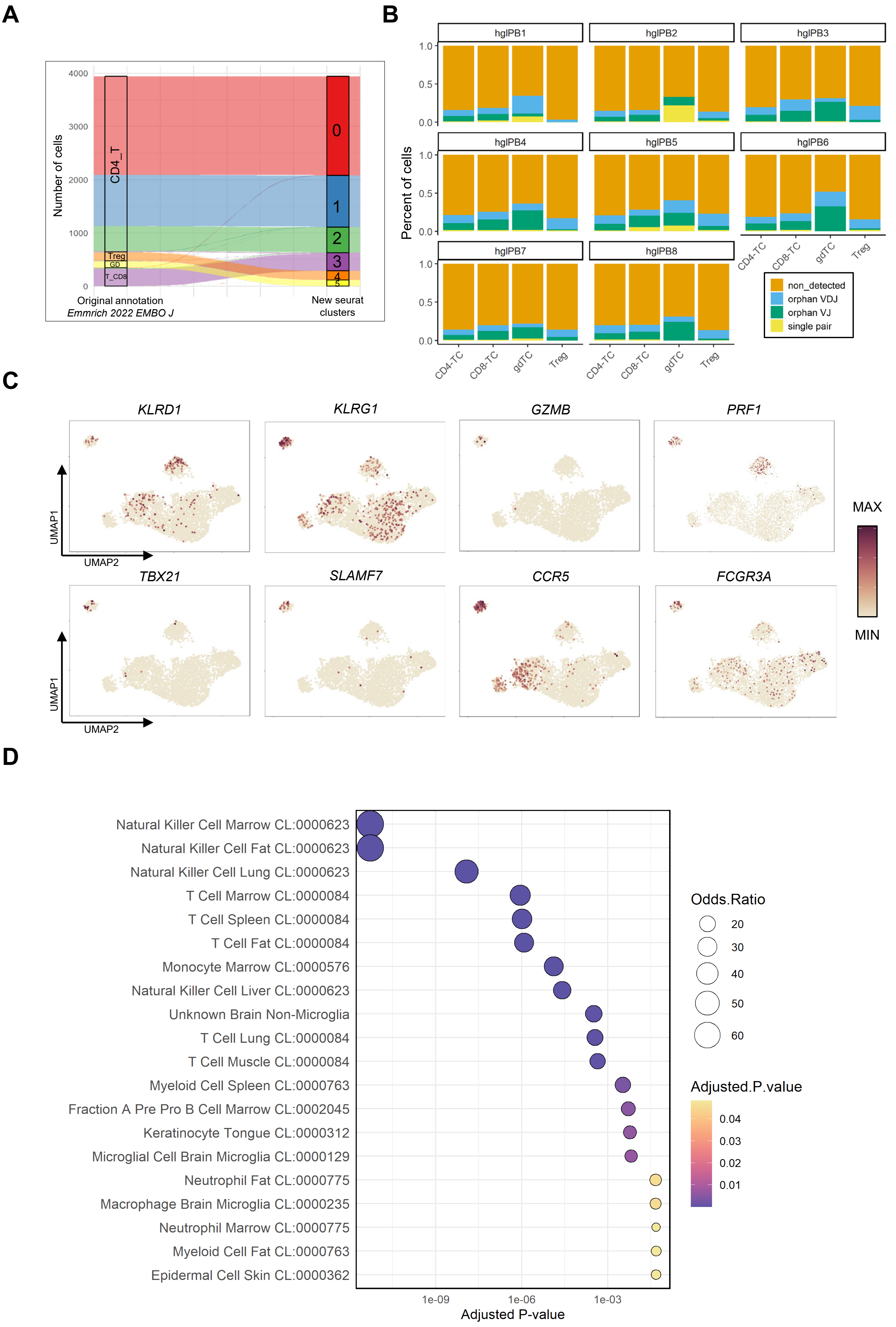

Supplementary Figure 7. (legend: see next page).

Supplementary Figure 7. Singe cell RNA and TCR-seq data from blood naked mole-rat T cells (I) (A) Alluvial plot indicating how the cluster IDs from the original study of the public single cell RNA-seq data[3] correlate with our new cluster re-annotation (Fig. 6A). (B) Bar plots indicating frequencies of detected TCR/CDR3 transcripts in the total T cell clusters (as identified in the original study, panel A). Orphan_VDJ = cells with single TRB or TRD chain, orphan VJ: cells with single TRA or TRB chain, single pair: cells with a single TRA-TRB or TRG-TRD pair, non_detected: cells where TRUST4 did not extract any CDR3 information (n=4 young-adult and 4 middle-aged adult animals) (C) UMAP plots displaying expression levels of selected genes. (D) Dot plot heatmap showing results of hypergeometric testing on the DGE of cluster 5 (Figure 6A) using gene sets from Tabula Muris database as a reference. The size of the dots represents Odds ratio while color illustrates the level of the p adjusted values (Benjamin-Hochberg correction). Source data are provided as a Source Data file.

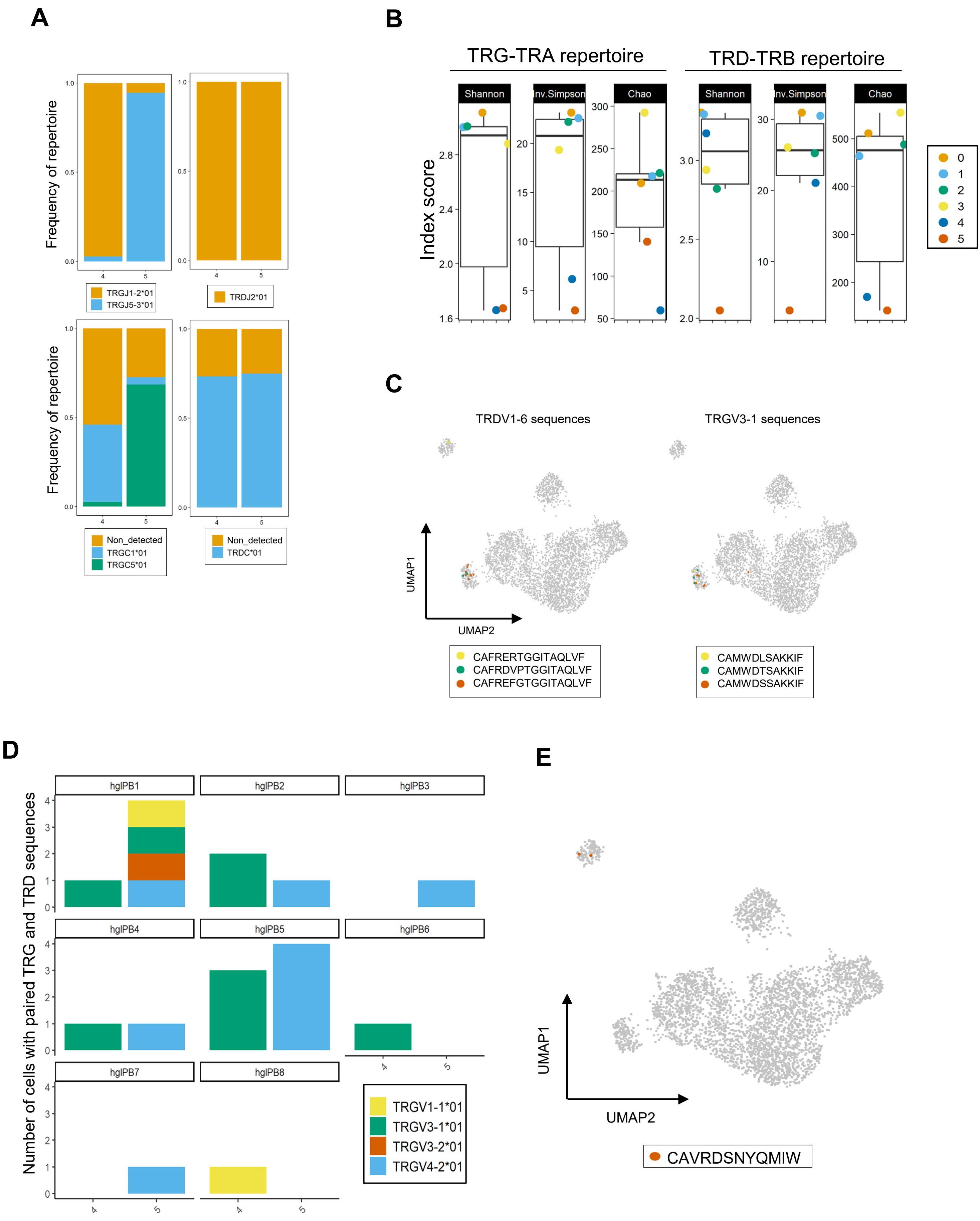

Supplementary Figure 8. (legend: see next page).

Supplementary Figure 8. Singe cell RNA and TCR-seq data from blood naked mole-rat T cells (II). (A) Bar plots of TRG/TRD J usage (top) and TRG/TRD C usage from $\gamma\delta$-enriched single cell clusters identified in Fig. 6A. Cells are derived from 4 young-adult and 4 middle-aged adult animals. (B) Diversity indices obtained from TRA/TRG (left) or TRB/TRD (right) repertoire of the different blood single cell clusters identified in Fig. 6A. (C) UMAP plots (as generated in Fig. 6A) highlighting the top 3 most abundant *TRDV1-6* (left) and *TRGV3-1* sequences (middle) from the single cell blood dataset. (D) Bar plot indicating the TRGV gene and the number of clonotypes present in the TRDV1-4- (cluster 5 of Fig. 6A) and TRDV1-6- (cluster 4 of Fig. 6A) enriched clusters in the distinct animals (each graph shows the data of a single animal). (E) UMAP plot (as generated in Fig. 6A) highlighting the TRAV1-TRAJ33 MAIT-like sequence. Source data are provided as a Source Data file.

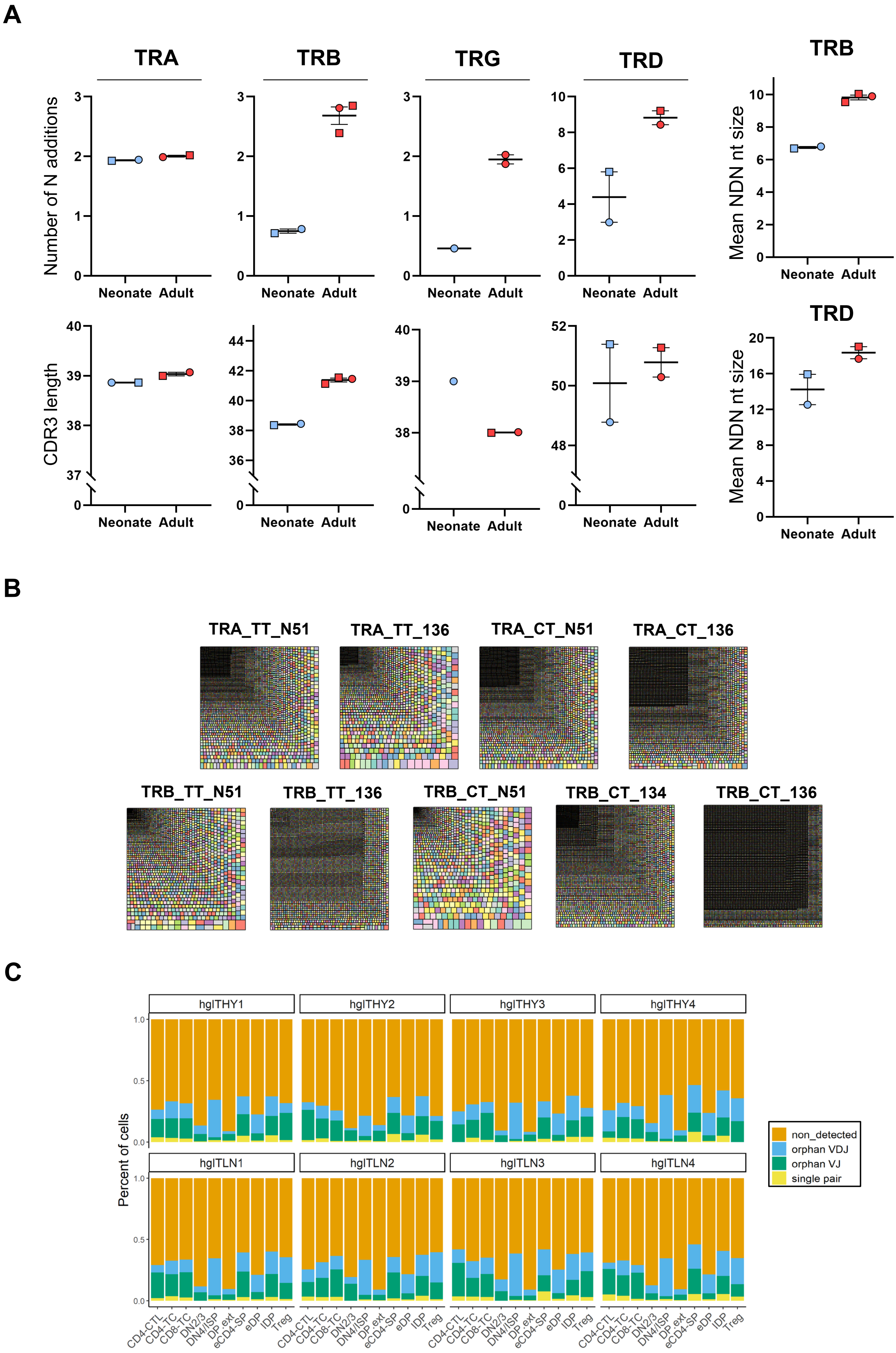

Supplementary Figure 9. (legend: see next page).

Supplementary Figure 9. Bulk and single cell TCR seq results from thymic samples. (A) Dot plots showing general CDR3 properties of CDR3α, β, γ and δ repertoires from neonate (n=1-2) and adult (n=2-3) thymic samples as obtained by bulk TCR sequencing. (B) Tree map overview from thymic TRA and TRB repertoires of all naked mole-rats (bulk TCR seq). See **Supplementary Table 1** for more information regarding each sample ID. (C) Bar plots indicating frequencies of detected TCR transcripts in the total thymocyte clusters (single cell seq). Orphan_VDJ = cells with single TRB or TRD chain, orphan VJ: cells with single TRA or TRB chain, single pair: cells with a single TRA-TRB or TRG-TRD pair, non_detected: cells where TRUST4 did not extract any CDR3 information. Thoracic (hglTHY) and cervical thymus (hglTLN) from each animal (4 in total) were processed separately. (A) Squares = cervical thymus samples, Circles = thoracic thymus samples. DN2/3 (double negative); DN4/ISP (immature single positive); eDP (early double-positive), lDP (late double-positive), eCD4-SP (early CD4 single-positive); CD4-TC ($CD4^+$ T-lymphocyte); CD8-TC ($CD8^+$ T-lymphocyte); DP.ext (extrathymic/circulating DPs); Treg (regulatory T cell); CD4-CTL ($CD4^+$ cytotoxic T-lymphocyte). Source data are provided as a Source Data file.

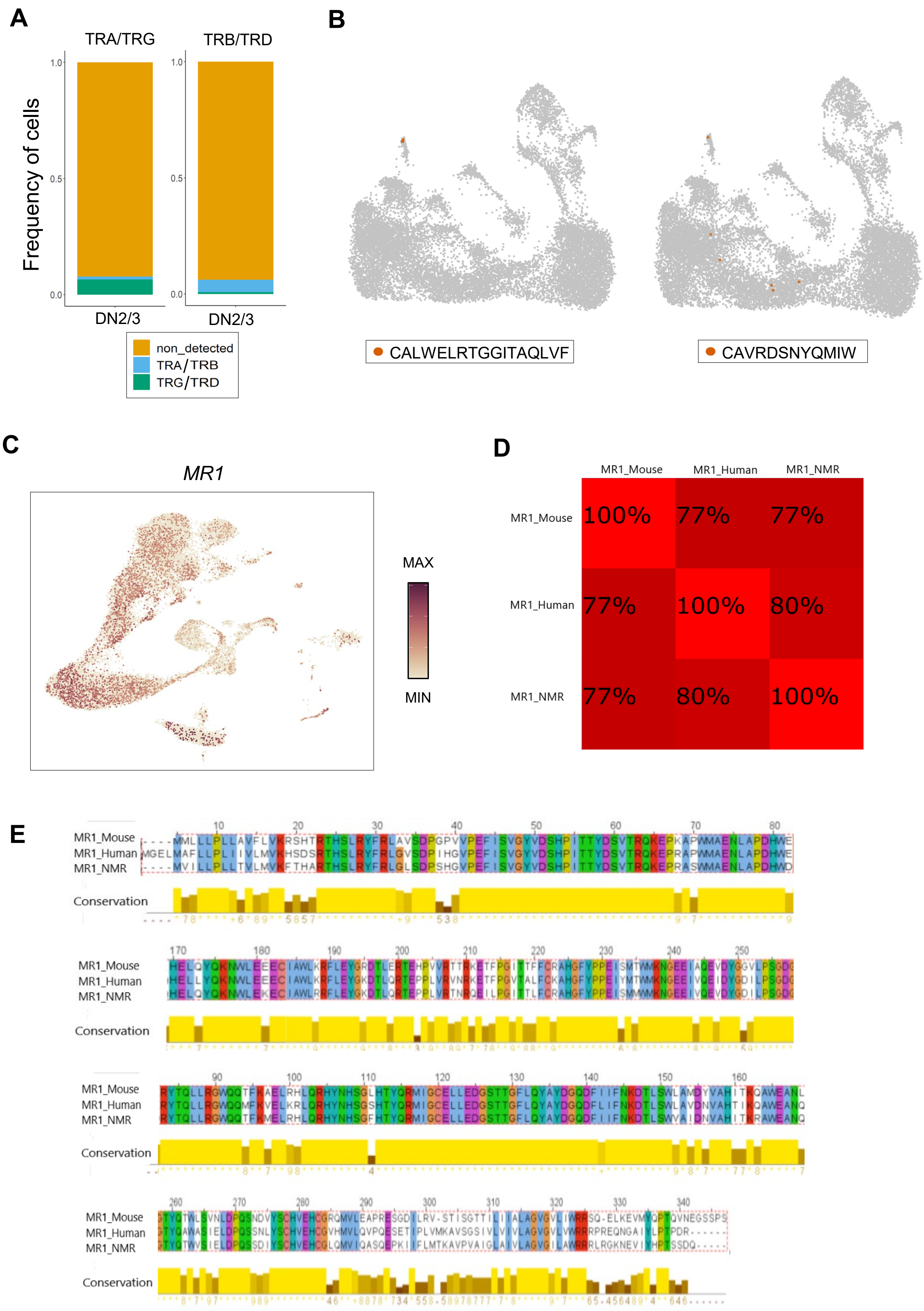

Supplementary Figure 10. (legend: see next page).

Supplementary Figure 10. Single cell RNA- and TCR-seq results from thymic samples and additional phylogenetic analysis. (A) Bar plot indicating the frequency of TRA/G (left) and TRB/D (right) in the immature DN2/3 cluster. non_detected: cells where TRUST4 did not extract any CDR3 information. Data is derived from 4 animals (2 young-adults and 2 middle-aged adults). (B) UMAP plots (as generated in Fig. 8C) highlighting the invariant TRDV1-4 (left) and TRAV1-TRAJ33 MAIT-like (right) sequences. (C) UMAP plots (as generated in Fig. 8C) from total cells from[4] indicating the levels of expression of *MR1* genes. (D) Matrix score calculation from alignment of *MR1* genes from human (*Homo sapiens*), mouse (*Mus musculus*) and naked mole-rat (*Heterocephalus glaber*). (E) Multiple sequence alignment of *MR1* genes from human (*Homo sapiens*), mouse (*Mus musculus*) and naked mole-rat (*Heterocephalus glaber*). Source data are provided as a Source Data file.

sample_ID	sample_ID_alt	ontogeny	age (days)	sex	CDR3a_reads	CDR3a_diversity	CDR3b_reads	CDR3b_diversity
**PB_neonate1**	**PB_N51**	neonate	0	unk	3981	42	12157	165
**PB_neonate2**	**PB_N52**	neonate	0	unk	7849	207	16142	778
**PB_neonate3**	**PB_N53**	neonate	0	unk	12308	253	22045	880
**PB_adult1**	**PB_134**	young-adult	831	♀	26213	2979	49917	4277
**PB_adult3**	**PB_136**	young-adult	831	♀	20169	5047	24261	13790
**PB_adult7**	**PB_001**	iddle-age adu	4305	♂	21696	1891	28264	7778
**SP_adult1**	**SP_134**	young-adult	831	♀	NS	NS	44513	1317
**SP_adult2**	**SP_135**	young-adult	831	♂	16653	5297	26223	15729
**SP_adult3**	**SP_136**	young-adult	831	♀	NS	NS	NS	NS
**SP_adult4**	**SP_137**	young-adult	1101	♂	14451	2655	17598	8017
**SP_adult5**	**SP_138**	young-adult	839	♀	10724	2341	16834	5567
**SP_adult6**	**SP_139**	young-adult	839	♂	11025	1833	24324	8579
**CT_neonate1**	**CT_N51**	neonate	0	unk	25282	2781	68778	1396
**CT_adult1**	**CT_134**	young-adult	831	♀	NS	NS	32941	7699
**CT_adult3**	**CT_136**	young-adult	831	♀	19819	10450	18731	16747
**TT_neonate1**	**TT_N51**	neonate	0	unk	26992	6145	8213	6439
**TT_adult3**	**TT_136**	young-adult	831	♀	25411	7860	54831	2802
**sample_ID**	**sample_ID_alt**	**ontogeny**	**age (days)**	**sex**	**CDR3g_reads**	**CDR3g_diversity**	**CDR3d_reads**	**CDR3d_diversity**
**PB_neonate1**	**PB_N51**	neonate	0	unk	145	4	72	17
**PB_neonate2**	**PB_N52**	neonate	0	unk	581	17	465	76
**PB_neonate3**	**PB_N53**	neonate	0	unk	190	10	387	44
**PB_adult1**	**PB_134**	young-adult	831	♀	7738	39	8053	145
**PB_adult3**	**PB_136**	young-adult	831	♀	2473	76	5303	221
**PB_adult7**	**PB_001**	iddle-age adu	4305	♂	125	22	1344	113
**SP_adult1**	**SP_134**	young-adult	831	♀	NS	NS	13286	61
**SP_adult2**	**SP_135**	young-adult	831	♂	1317	38	11836	419
**SP_adult3**	**SP_136**	young-adult	831	♀	13252	148	5656	281
**SP_adult4**	**SP_137**	young-adult	1101	♂	5395	103	3022	176
**SP_adult5**	**SP_138**	young-adult	839	♀	7058	132	6503	335
**SP_adult6**	**SP_139**	young-adult	839	♂	5262	78	1670	71
**CT_neonate1**	**CT_N51**	neonate	0	unk	NS	NS	2620	13
**CT_adult1**	**CT_134**	young-adult	831	♀	NS	NS	NS	NS
**CT_adult3**	**CT_136**	young-adult	831	♀	7758	567	5545	1250
**TT_neonate1**	**TT_N51**	neonate	0	unk	12147	11	14237	32
**TT_adult3**	**TT_136**	young-adult	831	♀	3615	271	2173	387

Supplementary Table 1, Bulk TCR-seq sample information. Each row represents a unique naked mole-rat. CDR3 reads column corresponds to the total amount of reads that could be mapped to the specific TCR locus. CDR3 diversity column indicates the total amount of detected clonotypes (at nucleotide level) in the sample. Unk = unknown, NS: no sequencing data.

sample id	total CDR3 reads	TRDV1-4	TRDV1-5	TRDV1-6	TRDV3	TRDV5
**CT_136**	5545	1081	147	4164	117	36
**CT_N51**	2620	1926	0	509	185	0
**LN_134**	987	521	5	460	1	0
**PB_001**	1344	270	0	1074	0	0
**PB_134**	8053	2631	6	5336	80	0
**PB_136**	5303	1875	34	3367	27	0
**PB_N51**	72	37	0	35	0	0
**PB_N52**	465	249	2	214	0	0
**PB_N53**	387	245	0	142	0	0
**SP_134**	13286	2803	0	10318	165	0
**SP_135**	11836	4860	47	6929	0	0
**SP_136**	5656	1552	21	4079	1	3
**SP_137**	3022	1415	0	1588	7	12
**SP_138**	6503	2467	9	4026	0	1
**SP_139**	1670	941	13	711	5	0
**TT_136**	2173	548	94	1471	44	16
**TT_N51**	14237	4052	0	10185	0	0

sample id	total CDR3 reads	TRGV1-1	TRGV3-1	TRGV3-2	TRGV4-2
**CT_136**	7758	46	12	80	7620
**CT_N51**	4600	0	0	0	4600
**LN_134**	4264	13	14	21	4216
**PB_001**	125	2	6	0	117
**PB_134**	7738	136	120	0	7482
**PB_136**	2473	31	41	13	2388
**PB_N51**	145	0	0	0	145
**PB_N52**	581	1	2	0	578
**PB_N53**	190	0	0	0	190
**SP_137**	5395	10	12	1	5372
**SP_138**	7058	25	12	13	7008
**SP_139**	5262	24	7	3	5228
**TT_136**	3615	13	30	34	3538
**TT_N51**	12147	882	0	378	10887
**SP_135**	1317	24	9	18	1266
**SP_136**	13252	62	0	108	13082

Supplementary Table 2. Number of CDR3 reads divided according to TRDV and TRGV usage in RNA transcripts. See **Supplementary Table 1** for more information regarding each sample ID.

Supplementary References

1. Katoh, K. & Standley, D. M. MAFFT Multiple Sequence Alignment Software Version 7: Improvements in Performance and Usability. *Molecular Biology and Evolution* **30**, 772–780 (2013).

2. Letunic, I. & Bork, P. Interactive Tree Of Life (iTOL) v5: an online tool for phylogenetic tree display and annotation. *Nucleic Acids Res* **49**, W293–W296 (2021).

3. Emmrich, S. *et al*. Characterization of naked mole-rat hematopoiesis reveals unique stem and progenitor cell patterns and neotenic traits. *EMBO J* **41**, e109694 (2022).

4. Emmrich, S. *et al*. Ectopic cervical thymi and no thymic involution until midlife in naked mole rats. *Aging Cell* **20**, e13477 (2021).
