# Supplementary material for: Invariant γδTCR natural killer-like effector T cells in the naked mole-rat
Source: Nat Commun. 2024 May 18;15:4248. doi: 10.1038/s41467-024-48652-z (PMC11102460; doi:10.1038/s41467-024-48652-z)
Supplement: Supplementary file 3 — Description of additional supplementary files [file 41467_2024_48652_MOESM3_ESM.pdf]

Description of Additional Supplementary Files

Supplementary Data 1: Differential expressed genes from clusters identified in the blood single cell dataset.

Differential gene expression analysis comparing gene expression of each cluster to all the others was performed by the ´FindAllMarkers' function using Wilcoxon-Rank sum test method (two-sided). DEGs were selected based on a log2-fold change (logFC) ≥ 0.2 difference between the average gene expression of the clusters, a percentage of expression equal to or greater than 15% in at least one test cluster (min.pct ≥ 0.15), a difference equal to or greater than 15% in the fraction of detection between the two groups (min.diff.pct ≥ 0.15) and adjusted p-value <0.05 (based on Bonferroni correction using all genes in the dataset).

Supplementary Data 2: Differential expressed genes from clusters identified in the spleen single cell dataset.

Differential gene expression analysis comparing gene expression of each cluster to all the others was performed by the ´FindAllMarkers' function using Wilcoxon-Rank sum test method (two-sided). DEGs were selected based on a log2-fold change (logFC) ≥ 0.2 difference between the average gene expression of the clusters, a percentage of expression equal to or greater than 15% in at least one test cluster (min.pct ≥ 0.15), a difference equal to or greater than 15% in the fraction of detection between the two groups (min.diff.pct ≥ 0.15) and adjusted p-value <0.05 (based on Bonferroni correction using all genes in the dataset).

Supplementary Data 3: Differential expressed genes from clusters identified in the thymus (thoracic and cortical) single cell dataset.

Differential gene expression analysis comparing gene expression of each cluster to all the others was performed by the ´FindAllMarkers' function using Wilcoxon-Rank sum test method (two-sided). DEGs were selected based on a log2-fold change (logFC) ≥ 0.2 difference between the average gene expression of the clusters, a percentage of expression equal to or greater than 15% in at least one test cluster (min.pct ≥ 0.15), a difference equal to or greater than 15% in the fraction of detection between the two groups (min.diff.pct ≥ 0.15) and adjusted p-value <0.05 (based on Bonferroni correction using all genes in the dataset).
